# Evidence for a high mutation rate at rapidly evolving yeast centromeres

**DOI:** 10.1186/1471-2148-11-211

**Published:** 2011-07-18

**Authors:** Douda Bensasson

**Affiliations:** 1 Faculty of Life Sciences, University of Manchester, Manchester, UK

## Abstract

**Background:**

Although their role in cell division is essential, centromeres evolve rapidly in animals, plants and yeasts. Unlike the complex centromeres of plants and aminals, the point centromeres of *Saccharomcyes *yeasts can be readily sequenced to distinguish amongst the possible explanations for fast centromere evolution.

**Results:**

Using DNA sequences of all 16 centromeres from 34 strains of *Saccharomyces cerevisiae *and population genomic data from *Saccharomyces paradoxus*, I show that centromeres in both species evolve 3 times more rapidly even than selectively unconstrained DNA. Exceptionally high levels of polymorphism seen in multiple yeast populations suggest that rapid centromere evolution does not result from the repeated selective sweeps expected under meiotic drive. I further show that there is little evidence for crossing-over or gene conversion within centromeres, although there is clear evidence for recombination in their immediate vicinity. Finally I show that the mutation spectrum at centromeres is consistent with the pattern of spontaneous mutation elsewhere in the genome.

**Conclusions:**

These results indicate that rapid centromere evolution is a common phenomenon in yeast species. Furthermore, these results suggest that rapid centromere evolution does not result from the mutagenic effect of gene conversion, but from a generalised increase in the mutation rate, perhaps arising from the unusual chromatin structure at centromeres in yeast and other eukaryotes.

## Background

Centromeres form the points at which the spindle attaches to DNA to ensure its proper segregation during cell division. This function is conserved from yeast to humans, and yet centromeres evolve rapidly [1-8]. Indeed, some have proposed that rapid centromere evolution could drive speciation [1,6,8]. More specifically, Henikoff et al [1] propose that because centromeres and the genes encoding their associated proteins are essential and more rapidly evolving than other DNA, their divergence is more likely than other DNA to result in genetic incompatibilities in hybrids following reproductive isolation.

Why would centromere sequences that are essential to proper chromosome segregation be evolving so fast? Most types of centromere are not defined by their DNA sequence [8], so a trivial explanation is that their rapid evolution is simply a consequence of low levels of selective constraint. However, there is growing evidence that centromeres evolve more rapidly even than selectively unconstrained parts of the genome [5,6], requiring more complex scenarios to explain this paradox of centromere evolution.

Several hypotheses have been put forth to explain fast centromere evolution. First, centromere sequences may act as selfish elements in the asymmetric meioses of female plants and animals [1,8]. Under this model of meiotic drive ("centromere drive"), centromere sequences have the potential to mutate in such a way that new alleles could bias their own segregation into an egg, and so centromeres evolve rapidly as a result of repeated selective sweeps as such alleles drive to fixation [1,8]. Alternatively, gene conversion could lead to the diversification of repeats [9] and thus facilitate rapid centromere evolution since most centromeres are repetitive [10,11]. Thirdly, centromeres may simply suffer a higher rate of mutation than other parts of the genome [5].

**Figure 1:**
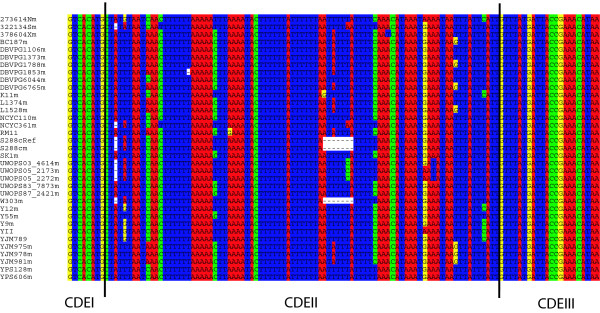
**Alignment of CEN4 for all strains analysed**. Centromeres are made up of two binding sites (CDEI and CDEIII) that are separated by an 87 bp CDEII. This CDEII region shows levels of polymorphism (θ_W _= 0.04) that are typical of CDEII in general, and many more point substitutions (N = 16) than would be expected for a transposable element fragment of the same length (N = 3). This alignment is unambiguous and so shows that the high rates of mutation at centromeres are not the result of alignment error or slippage mutations.

While rapid centromere evolution is observed in a diversity of species [3-8], alternative theories cannot explain rapid centromere evolution equally well in all eukaryotes. For example, yeasts have symmetric meioses, and thus there is no obvious advantage to meiotic drive [2,12]. Likewise, the point centromeres of *Saccharomyces *yeasts are short, with their rapidly evolving core (CDEII) spanning a non-repetitive region of only 80-90 bp (Figure 1). Thus, gene conversion involving repetitive DNA cannot directly explain rapid centromere divergence in yeasts. However, gene conversion is mutagenic in yeast [13], and so a high rate of gene conversion induced mutation may explain rapid evolution even in yeast centromeres. *Saccharomyces *yeasts therefore offer a powerful system with which to resolve the different forces governing fast centromere evolution.

In this study I address the causes of rapid centromere evolution in yeast by resequencing all point centromeres from 32 strains of *Saccharomyces cerevisiae *and studying published genomic data [14] for these strains and a further 34 strains of *Saccharomyces paradoxus*, to show that centromere evolution is rapid in both species. Using the population genetic data for *S. cerevisiae*, I test for the signatures of natural selection or recombination expected under meiotic drive or gene conversion models of rapid centromere evolution. I also estimate the mutation spectrum at centromeres and compare it with the spectra expected under spontaneous mutation [15] or mutagenic gene conversion [13]. Evidence presented here suggests that rapid centromere evolution in *S. cerevisiae *is due to a generalised increase in the mutation rate and not due to recombination or meiotic drive.

## Results

### Rapid centromere evolution in *Saccharomyces *yeasts

The point centromeres of *Saccharomyces *yeasts are made up of three Centromere DNA Elements (CDEI, CDEII and CDEIII). CDEI (8 bp) and CDEIII (25 bp) are protein-binding sites whose DNA sequence is highly conserved to preserve their function [16,17]. These are separated by CDEII, which is an AT-rich region of conserved length and base composition, but not primary sequence [17] (Figure 1). Consistent with a lack of any constraint on primary sequence, targeted resequencing of five CDEII sequences in *S. paradoxus*, the closest relative of *S. cerevisiae*, showed that these evolve more rapidly than other parts of the genome [5]. The limited sample of centromeres used leaves open the question of whether fast centromere evolution is a genome-wide phenomenon.

Here I show through analysis of whole-genome shotgun data from Liti et al (2009) [14] that rapid CDEII sequence evolution extends to at least 15 of 16 *S. paradoxus *centromeres (Table 1). *S. paradoxus *exist in diverged populations from Europe, Far East and America (formerly *S. cariocanus*) with little or no gene flow [5] and some reproductive isolation between them [18]. Levels of sequence divergence in CDEII are similar (e.g. Europe vs Far-East 8.4%, Table 1) to past estimates from five centromeres (12.9%) and much higher than past chromosome-wide estimates of divergence (1.5%), or even of unconstrained synonymous sites (4.7%) or transposable element (TE) fragments (4.6%) [5]. TE fragments have no obvious function and so are expected to evolve under no selective constraint, and in *S. paradoxus *TEs do evolve at the rate expected from synonymous sites in the absence of codon usage bias [5]. Direct comparison between CDEII and TE sequences in this study also shows that CDEII sequences diverge more rapidly and show higher polymorphism than these unconstrained TEs (Table 1), confirming previous results [5].

**Table 1:** Levels of polymorphism and divergence in *S. paradoxus *are lower for transposable elements than for CDEII

	CDEII loci	Median π or d	**95% C.I**.	TE loci	Median π or d	**95% C.I**.	CDEII:TE
Europe π	15	0.0066	0.0021-0.013	396	0.0017	0.0013-0.002	3.9
Europe- Far East d	15	0.084	0.059-0.10	339	0.028	0.026-0.030	3
Europe-American d	13	0.22	0.2-0.27	196	0.084	0.078-0.092	2.6

Table showing nucleotide diversity (π) within the European population or pairwise distance (d) between populations. 95% confidence intervals (C.I.) of each median are based on 10,000 bootstrap replicates. DNA sequence data are those published in Liti et al [14] and only 13-15 loci were available for *S. paradoxus *because PALAS alignment data were missing for all strains of *S. paradoxus *for CEN8 and because of low coverage of the American strains for CEN7, CEN8 and CEN12.

Fast centromere evolution may transcend species boundaries, since the rate of divergence between *S. cerevisiae *and *S. paradoxus *appears so high at centromeres that centromeres and their immediate flanks do not align (see Additional file 1, Figure S1), although most of the genome is readily aligned between the two species [19]. This is also the case when *S. cerevisiae *centromere loci are aligned with the outgroup species, *Saccharomyces mikatae *(see Additional file 1, Figure S2) [19], implying that rapid centromere divergence is not restricted to the *S. paradoxus *lineage.

Consistent with rapid centromere evolution, CDEII also has exceptionally high levels of variation in *S. cerevisiae*, resulting from point mutations scattered throughout the CDEII region (Figure 1). Mean nucleotide diversity in CDEII estimated from a global sample of strains (π = 0.04, 95% confidence interval from 10,000 bootstrap replicates: 0.034-0.046) is much higher than past genome-wide estimates of unconstrained nucleotide diversity in *S. cerevisiae *[20]. Indeed CDEII nucleotide diversity is higher than that for unconstrained non-coding sites (0.004, estimated from [20]) or for synonymous sites (0.007, estimated from [20]) (Wilcoxon signed rank tests, *P *= 3 × 10^-5^). Even the lowest estimate of nucleotide diversity observed across 16 centromeres (0.022) is 3-fold higher than these prior estimates for variation in unconstrained sequences. The higher variability in CDEII could be a consequence of a broader global population sample in the strains used here compared to those used by Doniger et al. [20]. When controlling for differences in sampling by comparing nucleotide diversity for CDEII to that estimated from TEs in the same strains using TE data from [14], I still see much higher nucleotide diversity in CDEII (Table 2 Figure 1).

**Table 2:** Mean nucleotide diversity (π) is higher at *S. cerevisiae *centromeres (in CDEII) than in selectively unconstrained sequences (TEs)

Population	Strains	CDEII S	CDEII π	TE S	TE π	P value	CDEII:TE
Global	34	222	0.04	1715	0.01	9 × 10^-11^	3.6
European	11	29	0.006	133	0.001	2 × 10^-10^	4.8
Sake	3	27	0.01	32	0.005	6 × 10^-6^	2.6
Malaysian	3	1	0.0005	0	0	0.02	n/a
Oak	2	0	0	0	0	n/a	n/a
West African	2	0	0	0	0	n/a	n/a

Nucleotide diversity of CDEII (π) was estimated across the CDEII component of all 16 centromeres and π for TEs was estimated for the 210 loci at which TEs appeared fixed. P values are the result of Wilcoxon tests comparing 16 estimates of π of CDEII to 210 estimates of π of TEs for each population.

The intergenic DNA flanking centromeres also has higher levels of variation (mean π = 0.01) than average unconstrained non-coding sites (0.004, Wilcoxon signed rank test, *P *= 9 × 10^-10^) [20]. This result is consistent with the finding that the intergenic DNA flanking centromeres shows more rapid evolutionary divergence than other intergenic regions in *S. paradoxus *[5], and the observation that centromere flanking DNA also fails to align between *Saccharomyces *species (see Additional file 1, Figures S1 and S2). This suggests that the phenomenon of rapid centromere evolution extends beyond the centromere core itself into the DNA immediately flanking centromeres. However, since CDEI and CDEIII are subject to selective constraint, and the intergenic regions flanking centromeres varying in length and constraint have been sampled to different extents, analyses presented here focus on CDEII. Furthermore, since more is known about the mutation process in *S. cerevisiae*, and since I have full coverage of centromere DNA sequences in *S. cerevisiae*, the analyses below are of *S. cerevisiae *data.

### A high mutation rate at *S. cerevisiae *centromeres

If centromeres evolve rapidly because repeated selective sweeps drive new centromere alleles to fixation, then we expect levels of polymorphism to be low at centromeres, even if overall centromere divergence is high. In contrast, if centromere evolution is rapid because of higher rates of mutation at centromeres, then we expect centromeres to be highly polymorphic within species as well as diverged between species. The high levels of polymorphism observed in *S. cerevisiae *and *S. paradoxus *are therefore more consistent with a high mutation rate underlying rapid centromere evolution.

The large number base pair substitutions causing high nucleotide diversity does not appear to result from the AT-richness of centromeres. Although yeast centromeres are a target for slippage mutations, insertions and deletions are not included in measures of polymorphism in this study (see Methods), and so these do not explain the high mutation rate at centromeres. While diversity is higher than expected compared to other regions it does not present a problem for DNA sequence alignment, and nucleotide substitutions are unambiguously scored (Figure 1). Furthermore, similarly AT-rich genomic regions away from the centromere do not show higher levels of divergence and polymorphism in *S. paradoxus *[5].

A drawback with using a global population sample of *S. cerevisiae *to assay levels of polymorphism, is that population structure exists within this global sample [14]. If new centromere alleles are driven to fixation by meiotic drive within subpopulations, divergence between subpopulations may lead to the inference of high levels of global polymorphism. To control for population structure, I examine levels of polymorphism within the populations defined by Liti et al [14]. In all three *S. cerevisiae *subpopulations where there are sufficient data, centromeres show significantly more nucleotide diversity than unconstrained TE sequences (Table 2). Likewise, analysis of *S. paradoxus *DNA sequence data also reveals high levels of polymorphism within populations (Table 1). This suggests that the rapid fixation of alleles within subpopulations expected from meiotic drive is not responsible for rapid centromere evolution in yeast. Indeed the rate of CDEII evolution is approximately 3 or 4 times higher than TEs whether this is estimated from diverged *S. paradoxus *lineages, or various *S. cerevisiae *and *S. paradoxus *populations (Tables 1 and 2). The constancy of this CDEII:TE ratio in both divergence and polymorphism comparisons is consistent with a neutral evolutionary force like mutation.

### Recombination does not explain rapid centromere evolution directly

Recent work suggests that recombination in the form of gene conversion may be an alternative mechanism to explain rapid centromere evolution [10]. Therefore, I tested for evidence of recombination in centromeres using a likelihood method [21] and the classic four-gamete test for recombination [22]. Given the high density of polymorphic sites within centromeres and the number of strains studied, some identical polymorphisms will have arisen on different haplotype backgrounds by chance. Under most tests of recombination these cases of homoplasy will be mistaken for recombination. The tests of recombination used here estimate the probability of recombination, given the likelihood of homoplasy [21] (see Methods, Supp Table 2 and Supp Table 3).

**Table 3:** Summary of possible recombination events in or near the centromere

Locus	R_min_	No. of Sites	Distance from centromere (bp)
CEN1	2	2	0 (within CDEII)
CEN2	4	7	< 10, 10-13, 229-281, 281-353
CEN11	2	5	< 51, 157-281
CEN14	1	3	14-1208

The recombination events listed here show statistically significant evidence (P < 0.05) for recombination using 1 or more test implemented in LDhat, as well as with the 4-gamete test followed by simulation to test the likelihood of multiple mutations. R_min_ is the minimum number of recombination events estimated at each locus [22], and No. of Sites is the number of sites showing evidence for recombination.

Overall, I find no evidence for recombination in *S. cerevisiae *centromeres, with only one possible exception (Table 3 Supp Table 2). In the one centromere that shows potential evidence for recombination, CEN1, the P-value is only marginally significant, and depends on only two segregating sites with evidence of either recombination or homoplasy. The removal of only one site is sufficient to remove the signal of recombination. In contrast, there is much stronger evidence for recombination in the regions flanking three out of sixteen centromeres (Table 3 Additional file 1, Table S3), and some of these events appear to occur very close to the centromere (Table 3).

### The mutation spectrum at centromeres is similar to genome-wide spontaneous mutation

To further resolve whether high levels of variation in centromeres result from the action of gene conversion or spontaneous mutation, I investigated the mutation spectrum in centromere sequences. The data presented here are of sufficient quality for this purpose, since I expect few or no sequencing errors in the *S. cerevisiae *centromere data (estimated error rate: < 3 sequencing errors total, see Additional file 1, Supplementary text). In order to distinguish insertions from deletions and to infer the direction of change of base pair substitutions from polymorphism data, we need to know the ancestral state at each site. This inference is complicated by the fact that no useful outgroup is known for *S. cerevisiae *centromeres since *S. paradoxus *is too diverged at centromeres to align CDEII (Additional file1). Therefore I examined the mutation spectrum of 114 base pair substitutions, and 22 indels that are each unique to one haplotype and therefore assumed to exist in the derived state. Approaches that use gene genealogies or phylogenetic trees to infer the polarity of mutations include unique as well as other alleles as derived, and thus this method is conservative in terms of identifying unambiguously polarisable mutations.

In CDEII, transitions are as common as transversions, given the fact that there are two times as many possible transversions as transitions (46:68, Table 4, Binomial exact test, *P* = 0.11). A lack of transition bias is also the case for genome-wide estimates (12:19, Table 4, Binomial exact test, *P* = 0.57) [15], and when all data on wild-type patterns of spontaneous mutation are pooled (142:252, Table 4, Binomial exact test, *P* = 0.26) [13,15,23]. Moreover the transition:transversion ratio in CDEII is not significantly different from that seen from pooled counts of spontaneous mutations in wild-type cells in *S. cerevisiae *(Fisher's exact test, *P* = 0.4). In contrast, transitions are more common than transversions when levels of gene conversion are high (32:24, Table 4, [13]) but this transition bias is not observed in centromeres (Fisher's exact test, *P* = 0.049). Thus, the ratio of transitions to transversions observed in CDEII is like those generally observed for spontaneous mutation and not like that of gene conversion in *S. cerevisiae *(Table 4).

**Table 4:** Mutation spectrum at centromeres is unlike that of gene conversion

Study	Ts: Tv	Ts:Tv freq	1 bp deletions	Del:BPS freq	1 bp insertions	Other
CDEII	46: 68	0.68	4	0.047	0	18^a^
Genome-wide (WT)	12: 19	0.63	1	0.032	0	1
URA3 Lang (WT)	46: 121 *	0.38	22	0.017	3	15
CAN1 Lang (WT)	65: 85	0.76	56	0.034	8	13
URA3 Hicks (WT)	19: 27	0.70	5	0.014	1	9
URA3 Hicks (GC)	32: 24 *	1.33	32	0.074	1	14

The mutation spectrum of CDEII is compared to the wild type spontaneous mutation (WT) inferred from various studies (Genome-wide, [15]; Lang, [23]; Hicks, [13]), and the mutation spectrum expected under gene conversion (GC, [13]). "Other" indicates indels that are -> 1 bp long, and in the case of CDEII also includes indels occurring in homopolymer runs that were 5 bp or longer (N = 14), other mutations described as "Other" for the WT and GC datasets are described in [13]. The deletion: base pair subsitution frequency (Del:BPS) corrects for the difference in target length at which deletions could be observed compared to the target length for base pair substitutions ([23], e.g. for URA3, indel target size = 804 bp, and BPS target size = 104 bp). For CDEII the number of deletions (N = 4) was compared to the number of base pair substitutions (N = 84) outside homopolymer runs. * Significantly different from CDEII (Fisher's exact test, P < 0.05). Poisson tests showed that none of the Del:BPS frequencies were significantly different from that of CDEII.

**Figure 2:**
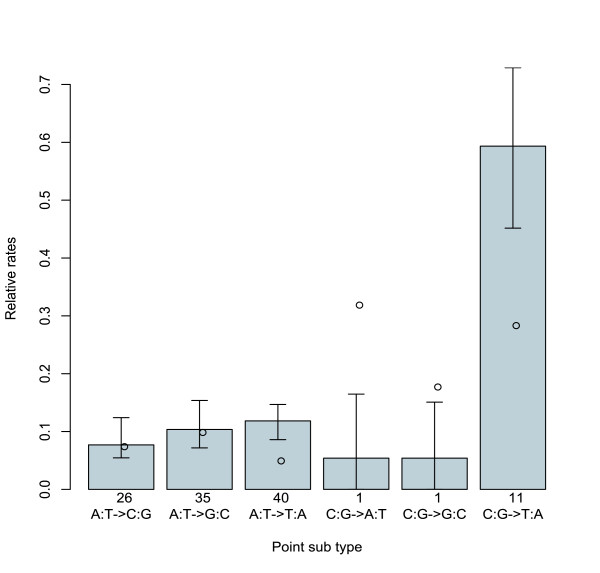
**The point substitution spectrum in CDEII**. Relative rates of each point substitution (r_i-> j_) are counts of substitutions from i to j (n_i-> j_) given the total number of substitutions (∑ n_i-> j_), normalised for the base composition (p_i_, p_AT _= 0.95 in CDEII) and then rescaled so that totals add up to one: r_i-> j _= ((n_i-> j_/∑ n_i-> j_)/p_i_)/∑((n_i-> j_/∑ n_i-> j_)/p_i_). Error bars are 95% confidence intervals estimated from 1000 bootstrap replicates in which samples of 16 centromeres were sampled at random with replacement for each replicate using R. Open circles show the relative rates of spontaneous mutations observed genome-wide [15]. The genome-wide estimates are from a total of 31 point substitutions, and the errors associated with these are therefore too large to show here. Total counts of each point substitution type (n_i-> j_) observed in CDEII are shown in the lower margin.

There is also no significant difference in the mutation spectrum seen at centromeres compared to that seen genome-wide [15] (GLM, d.f. = 5, *P* = 0.09, Figure 2). There is however a significant bias for C-> T or G-> A transitions (C:G-> T:A, in Figure 2, GLM, d.f. = 1, *P* = 9 × 10^-^^6^), suggesting that cytosine deamination is a common source of mutation at centromeres. High levels of C:G-> T:A were also noticed and discussed in the genome-wide study of mutation, along with levels of C:G-> A:T transversion that may possibly be elevated due to the conversion of guanine to 8-oxo-guanine [15]. CDEII is too AT-rich for an accurate assessment of the levels of C:G-> A:T transversion (Figure 2).

Apart from an unusually high transition bias, another signature of gene conversion associated mutation is a high level of single nucleotide deletions relative to base pair substitutions [13], and so here I also examine deletions within CDEII. An important consideration when studying insertions or deletions (indels) in CDEII is its unusual homopolymer run content. Runs of As and Ts are of functional importance within CDEII [17], and such runs are known to lead to a much higher frequency of indels than in other sequence [15,24]. Thus there may be significant differences in the frequency of indels between CDEII and spontaneous or gene conversion mutation spectra, simply because of differences in the homopolymer run content in the types of sequence studied. There are indeed many more homopolymer runs of 5 bases or more in CDEII sequences (72 in 1371 bp) compared with the URA3 sequence of *Kluyveromyces lactis*, which was used by Hicks et al. [13] to characterise the mutation spectrum under gene conversion (0 in 804 bp), (Poisson test, *P* = 5 × 10^-15^). To control for this fundamental difference in sequence composition, indels occurring inside homopolymer runs of 5 bp or longer are considered separately (Table 4).

Although the frequency of single nucleotide deletions in centromeres is not significantly different from that seen under gene conversion, it does appear more similar to that seen in surveys of spontaneous mutation in wild-type cells (Table 4). Deletions within CDEII are slightly higher than other estimates, but this may result from the prevalence of short homopolymer runs in CDEII. After exclusion of homopolymer runs of 5 nucleotides or more, more homopolymer runs that are 4 bp long remain in the CDEII data (N = 38), compared with URA3 (N = 12) (Poisson test, *P* = 0.003), and such runs may still attract a higher frequency of deletion. Overall, both the point and deletion mutation spectrum at centromeres is more like that expected under wild-type spontaneous mutation than that expected if the higher mutation rate at centromeres resulted from high levels of gene conversion (Table 4).

## Discussion

This study extends the past finding that *S. paradoxus *shows rapid evolution in five centromeres [5] to all centromeres of this species and to a second species with point centromeres, *S. cerevisiae *(Figure 1, Tables 1 and 2, Figures S1 and S2). *S. cerevisiae *is an especially useful species in which to study the cause of rapid centromere evolution because its point centromeres are easy to sequence and patterns of recombination and mutation within its genome are exceptionally well characterised [13,15,23,25,26]. The population and comparative analyses of centromere DNA sequences presented here lead to the conclusion that rapid centromere evolution is caused by a generalised increase in the mutation rate and not by meiotic drive or recombination.

Under the meiotic drive theory of rapid centromere evolution, we expect low polymorphism within centromeres. Point centromeres, the one type of centromere where this is easily and accurately assayed, show the opposite pattern; high diversity within species or populations (Table 1, Table 2). This is the case for at least three populations of *S. cerevisiae *(Table 2) and one population of *S. paradoxus *(Table 1, [5]). Whether levels of divergence or polymorphism are measured, CDEII seems to evolve 3 or 4 times faster than selectively unconstrained DNA (Tables 1 and 2). High levels of polymorphism suggest that rapid centromere evolution is not the result of the sweeps of natural selection predicted by meiotic drive. The exceptionally high levels of polymorphism seen here, and the constant level at which centromere polymorphism or divergence is increased relative to other parts of the genome, are expected if high mutation at centromeres causes their rapid evolution.

The proteins that bind to animal and plant centromeres also contain rapidly evolving regions, and this could be because of positive selection for mutations that suppress meiotic drive of centromeres during female meiosis [1,27]. In contrast, there is no such evidence of positive selection in the centromere binding proteins of yeast [27], and this is consistent with a high mutation rate as a mechanism for rapid centromere evolution in yeast. If there is no evidence for compensatory mutations in yeast centromere binding proteins, then perhaps this implies that the rapid divergence of CDEII sequences has no functional consequences. Experimental replacement of CDEII sequences with random sequence of the same length and base composition does however appear to increase rates of segregation distortion in *S. cerevisiae *[17]. Therefore it is possible that the high mutation rate at yeast centromeres has functional consequences, but these could only contribute to yeast speciation under a simple Dobzhansky-Muller model [28]: centromeres diverge so that they are no longer bound by their associated binding proteins, as opposed to a meiotic drive model for speciation [1,27] in which meiotic drive at centromeres and its suppression by centromere binding proteins leads to post-zygotic reproductive isolation.

Recent evidence suggests that gene conversion at centromeres could contribute to rapid centromere evolution in maize [10], leading to the proposal that this force could generally explain rapid centromere evolution in eukaryotes [10,11]. The findings of the study in maize came as a surprise, since it has long been thought that recombination is suppressed at centromeres [29], and this has been confirmed in yeast [30] and other species [11,31,32]). Using population data in yeast and some of the same approaches used for maize [10], I find evidence for recombination very close to centromeres though not within them (Table 3, see Additional file 1, Table S2, Table S3). A number of recombination events may have occurred close to CEN2 (Table 3), where a double-stranded break hotspot is also predicted [25]. High-resolution genome-wide mapping of the crossover and non-crossover events from a large number of meioses in *S. cerevisiae *also shows that crossovers sometimes occur close to centromeres, but not within them, and that gene conversion does not occur close to centromeres at all [26]. The absence of a detectable signature of recombination events within centromeres, together with the lack of an obvious mechanism by which gene conversion would increase diversity in non-repetitive point centromeres, suggests that gene conversion does not lead to rapid centromere evolution, at least in the way proposed in maize.

Gene conversion is mutagenic [13], so even if the signatures of gene conversion have been obscured in yeast centromeres, perhaps their high mutation rate does result from high rates of gene conversion as a result of this mutagenicity, if not as a result of the products of recombination. Analysis of the mutation spectrum at *S. cerevisiae *centromeres, suggests this too is not the case. The mutation spectrum in CDEII is more like that seen genome-wide and in wild-type strains in studies of spontaneous mutation than it is like the spectrum expected specifically under gene conversion (Table 4). Thus the rapid evolution of yeast centromeres may not rely on the action of a specific DNA repair system like that involved in gene conversion.

In summary, it appears that a generalised increase in the mutation rate is responsible for the rapid evolution seen at point centromeres, and this is not the result of gene conversion as recently proposed [10]. Given that rapid centromere evolution occurs in a broad range of eukaryotes [3-8], it is possible that high mutation rates could also contribute to the rapid evolution of other eukaryotic centromeres.

Apart from rapid evolution, another general characteristic of centromeres is that their DNA is wound round a histone specific to centromeres, CENH3 [8]. This leads to a nucleosome structure that is fundamentally different at centromeres compared to other parts of the genome [8,33,34]. There is evidence in yeast, human and fish that rates of evolution are higher in DNA that is bound in canonical nucleosomes than in flanking linker regions [35-38]. In addition, experimental studies on *S. cerevisiae *and human show increased mutation rates at nucleosomes because DNA repair proteins have reduced access to DNA once DNA is packaged on histones [39,40], so this may explain the elevated evolutionary rates observed for DNA in nucleosomes [36,37]. It may be especially difficult to unwind DNA from a relatively rare histone variant, such as CENH3, with an unusual nucleosome structure, and this could lead to inefficient DNA repair at centromeres. Similarly, the subtelomeres of *S. cerevisiae *show accelerated base-pair substitution and also have a non-canonical chromatin structure [41]. The alternative conformation of chromatin at centromeres may be necessary for centromere inheritance in the case of regional centromeres or more generally for centromere function [33], but may also expose centromere DNA to higher rates of mutation and sequence evolution.

## Conclusions

In this work I present a complete dataset of sequences for all 16 centromeres in 34 strains of the model yeast species, *Saccharomyces cerevisae*, including more than 400,000 nucleotides of centromeric DNA sequence. Using population genetics theory and methods to test for the past effects of natural selection and recombination at centromeres, I rule these forces out as major contributors to rapid centromere evolution in yeast. Moreover, as *S. cerevisiae *is also a model for the study of mutation, I compare the mutation spectrum at centromeres to those expected under different modes of DNA repair. These analyses collectively support a model of high mutation rate, rather than meiotic drive or gene conversion, as being the principal force driving rapid centromere evolution in yeast. Yeast centromeres are simpler than those of plants or animals and yet they have several characteristics in common with them, such as rapid centromere evolution and an unusual chromatin structure. The results from this study imply that other eukaryotes, such as animals and plants, probably also suffer a high rate of spontaneous mutation at their centromeres.

## Methods

### DNA sequencing of centromeres of *S. cerevisiae*

The DNA sequence data available from the *Saccharomyces *Genome Resequencing Project (SGRP) are only available at low genome coverage for most strains (between 1x and 3x). As a consequence, DNA sequence is only available at approximately 40% of centromere sites. This would yield too few data for a full analysis of recombination and mutation spectrum at centromeres. The *S. cerevisiae *strains used by the SGRP were therefore fully resequenced at centromeres for this study and the SGRP data were used to test DNA sequence quality (see Additional file 1).

Set 1 of SGRP strains were ordered from the National Collection of Yeast Cultures (NCYC, http://www.ncyc.co.uk/sgrp.html). All 36 strains provided by NCYC are the monosporic derivatives of the original parental strains sequenced as part of the SGRP. Thus they are expected to exist as fully homozygous diploids, with no ambiguous sequence resulting from heterozygosity, except perhaps at the MAT locus. These monosporic derivatives of their parents are described here using the name of the parental strain followed by an "m" (e.g. YS2m). During the course of this study, I found that four SGRP strains (YS2m, YS4m, YS9m and DBVPG6040m) show signs of heterozygosity at many sites even though they are monosporic derivatives. This suggests that they exist as polyploids or aneuploids and so they were excluded from all analyses presented here.

DNA was extracted for the 36 strains of *S. cerevisiae *in this set using the Wizard Genomic DNA purification kit from Promega, according to the manufacturer's instructions for yeast. DNA was diluted for each strain and the equivalent of 0.05 μl of extract (approximately 1 ng DNA) for each strain was used to amplify each of 16 centromere loci in 15 μl volumes by PCR: 1.5 mM MgCl_s_, 1 × Buffer, 0.2 mM each dNTP, 0.3 μM each primer, 1 unit BioTaq™ DNA polymerase (Bioline); Cycling conditions: 94°C 4 mins; 35 cycles: 94°C 40 secs, 55°C 1 min, 72°C 1 min 20 secs; 72°C 7 mins. PCR products were visualised on a 1% agarose gel and 5 μl from each was treated with ExoSAP-IT™ according to manufacturer's instructions (GE Healthcare) to degrade leftover dNTPs and single-stranded primers. Each PCR product was sequenced using at least two primers, one from each strand, on an ABI Prism 3100 Genetic Analyser. Primers were designed using primer3 (version 1.1.4, http://primer3.sourceforge.net), and a full list of the primers used for PCRs and sequencing is in Additional file 1, Table S1. Staden version 1.7.0 http://sourceforge.net/projects/staden/ was used to assign Phred (version: 0.020425.c) quality scores to reads, and to assemble a single consensus sequence for each centromere using the Gap4 assembler. A consensus quality threshold of Q40 was used throughout this work, and each consensus showed bases as ambiguous ("N") if their quality score was below this threshold. DNA sequences were aligned in SeaView 4.0 http://pbil.univ-lyon1.fr/software/seaview.html [42] against the reference strain sequence of S288c included with the SGRP data. The 509 DNA sequences generated, aligned and annotated as part of this study are available from NCBI [GenBank:HQ339369-HQ339877]. A comparison of these centromere DNA sequence data, to the SGRP data generated using a whole-genome shotgun sequencing approach showed no detectable errors due to *Taq *polymerase, DNA sequencing or base-calling errors (see Additional file 1 Supplementary Text).

### Whole-genome shotgun data for centromere and transposable element sequences in *S. cerevisiae *and *S. paradoxus*

The SGRP data were downloaded from ftp://ftp.sanger.ac.uk/pub/dmc/yeast/latest/ on 4th February 2009. Only actual (no imputed) data were used. The centromere for the reference strains of *S. cerevisiae *and *S. paradoxus *used in Liti et al. [14], and its component conserved DNA elements (CDEI, CDEII and CDEIII) were annotated using the consensus sequence motifs for CDEI and CDEIII described in Baker and Rogers [17], and a perl script CENannotate.pl (available on request).

The SGRP PALAS alignments analysed in Liti et al [14] for the 16 centromere loci of 32 strains of *S. cerevisiae *(for estimation of error rates) and all 35 strains of *S. paradoxus *were extracted using alicat.pl (a perl script provided with the SGRP data). A quality threshold of Q40 was used for the SGRP data, and sites with a lower quality score were represented with an "N".

Apart from the 32 *S. cerevisiae *strains that show no evidence of heterozygosity (see above), the publicly available genome sequences for two more strains, RM11.1a http://www.broadinstitute.org/annotation/genome/saccharomyces_cerevisiae/Home.html and YJM789 [43], were included in this and all subsequent analyses of *S. cerevisiae*. According to Liti et al. [14], six of the 36 *S. cerevisiae *SGRP strains may be identical clonemates of others in the data and they exclude these from their genome-wide analysis of nucleotide diversity. The centromere data presented here only supports this conclusion for 2 strains (NCYC110 and UWOPS05-217.3), and these are not excluded from the analysis. The Hawaiian strain of *S. paradoxus *(UWOPS91-917.1) does not belong to European, Far Eastern or American populations of *S. paradoxus *and so this strain is excluded, bringing the total number of *S. paradoxus *strains included in the analysis to 34.

Annotations of transposable elements (TEs) in the version of the *S. cerevisiae *(S288c) and *S. paradoxus *reference genomes against which all SGRP PALAS sequences are aligned, were produced using RepeatMasker and REANNOTATE [44] http://www.bioinformatics.org/reannotate. The size distribution of the resultant *S. cerevisiae *483 transposable element fragment annotations, were approximately as expected. Most fragments were less than 400 bp long (solo-LTR fragments) and there were a few TEs that were longer and are probably full-length elements or degenerated versions of them. There are 572 annotated transposable elements for *S. paradoxus*, and more than 200 of these are 300-400 bp in length, and so are probably recent single solo-LTR insertions. There are few fragments that are the expected size of full-length transposable elements, but this is most likely because the reference sequence used represents an incomplete genomic assembly and full-length transposable elements are where gaps in the assembly are most likely to arise.

SGRP PALAS alignments were extracted for each TE locus using alicat.pl (with a Q40 threshold), together with alignment corresponding to 100 bp of flanking reference sequence on either side of the TE. All alignments were inspected and each transposable element alignment was manually assigned as fixed, polymorphic or complex. Complex alignments are those in which fixed elements contain additional polymorphic transposable element insertions, or those in which the alignment does not extend unambiguously into the regions flanking the transposable element region. Fixed elements containing a polymorphic solo-LTR insertion that is present in only one strain are still included in the analysis because estimates of sequence divergence will not be affected by such events. In the case of *S. paradoxus*, if a polymorphic element occurs inside a fixed element, and the polymorphism is not present in European strains, then this fixed element is still included, since estimates of divergence between populations and European nucleotide diversity will not be affected by the polymorphism. In this way, 210 and 396 fixed TE fragments were identified in *S. cerevisiae *and *S. paradoxus*, respectively, for comparison to centromeres in their levels of polymorphism and divergence.

### Estimating levels of divergence and polymorphism

Population divergence, numbers of segregating sites and other measures of polymorphism for both *S. cerevisiae *and *S. paradoxus *were estimated for each fixed TE, CDEII and centromere flanking region, using Variscan 2.0 (http://www.ub.es/softevol/variscan/) [45]. Variscan is able to handle the large amount of missing data seen for the SGRP data, in estimating levels of polymorphism. Polymorphism levels were estimated from all sites where at least two valid DNA sequences were available (Variscan parameters: CompleteDeletion = 0, FixNum = 0, NumNuc = 2, see Variscan documentation for details). Insertions and deletions were treated as missing or ambiguous data and so estimates of polymorphism are not affected by the placement of these in the alignment or by slippage as a result of the prevalence of homopolymer runs within CDEII (Figure 1). Levels of polymorphism were estimated as both nucleotide diversity (average pairwise distance, π) and Watterson's theta (θ_W_) [46], but results were the same regardless of whether π or θ_W _were used to compare levels of polymorphism between centromeres and other regions. Subsequent statistical analyses were in R 2.9.0.

### Testing for recombination at centromere loci

Two approaches were used to test for recombination in the presence of potentially high rates of mutation. Firstly, the likelihood method based on coalescent theory developed by McVean et al. [21] was used together with their likelihood permutation tests that test for statistical significance. This is implemented by McVean et al. in LDhat, and was applied to each locus using the LDhat modules convert, pairwise, and lkgen. As in McVean et al. [21], sites with minor allele frequencies less than 0.1 were excluded. To account for the significantly higher levels of polymorphism within centromeres compared to their flanking regions, which are probably under some selective constraint, centromeres and flanking regions were analysed separately, with θ = 0.1 within centromeres and θ = 0.01 in flanking regions. LDhat was run under a crossing-over model, and then again using a gene conversion model (with the conversion tract length set to 100 bp). The two models gave similar results and the results under the gene conversion model are shown in Additional file 1, Supplementary Tables 2 and 3. Secondly, the four-gamete test was applied to every site in each locus to identify sites that can only be explained by a recombination event or homoplasy using a custom perl script fourgamete.pl (available on request). I then estimated the minimum number of sites showing homoplasy needed to explain the data, and tested the likelihood of the observed number of homoplasious sites with 1000 simulated replicates in R, given the length of the sequence and the number of segregating sites (homoplasysim.pl, available on request). This second approach was also applied separately for centromeres and flanking regions, since the random simulation used to test significance also assumes a uniform mutation rate. To test whether all evidence for recombination is successfully explained by the minimum set of homoplasious sites identified in this way, these sites were removed from each full alignment (centromeres with flanks), and the four gamete analysis was repeated on the resultant full alignments. There was no significant evidence for recombination after removal of the minimum set of sites with homoplasy. LDhat and the four-gamete test with significance tests gave consistent results. This double approach leads to a large number of statistical tests (see Additional file 1, Table S2 and Table S3), and so some significant results are expected by chance, in addition LDhat appears to be very sensitive to heterogeneity in the mutation rate leading to several false positives (Additional file 1, Table S2, highlighted in pink). Recombination was therefore only assumed for a locus when there was some evidence for this using both approaches.

### Characterising the mutation spectrum

In order to polarise point substitutions, I consider mutations that are unique to one haplotype to represent the derived allele. In each alignment I therefore count the number of each unique substitution type (e.g. A-> C, C-> G) denoted as n_i-> j _(e.g. n_A-> C_, n_C-> G_). Base composition is likely to differ among loci and CDEII is more AT rich than the genome-wide average, I therefore also consider the number of bases available for each type of change, denoted as α_i _(e.g. α_A_, α_C_). α_i _in turn is estimated as the sum of nucleotide (i) in all strains sequenced for that locus. Using the program unique.pl, each alignment was reduced to an alignment where each haplotype was only represented once by the strain with the longest unambiguous sequence belonging to that haplotype, n_i-> j _and α_i _were then estimated from the resultant DNA sequence alignment of each locus. To test for significant differences among CDEII loci in the point substitution spectrum or for significant point substitution biases in the total dataset, a generalised linear model (GLM) was fit to the data in R with Poisson errors. The number of point substitutions (n_i-> j_) was set as the response variable, with locus (CEN1 to CEN16), and unique substitution type as explanatory variables and with α_i _as offsets. The fit of each model to its assumptions was checked and simplified according to the recommendations in Crawley [47]. The centromere data were also compared to the spectrum of the 31 point substitutions identified in the genome-wide analysis using a GLM with Poisson errors.

Unique indels and the length of sequence in which they occur were also summarised using the program unique.pl. Alignments were first reduced by unique.pl so that each haplotype is only represented once and unique changes are again assumed to represent the derived state. Indels occurring inside homopolymer runs of 5 bp or longer are considered separately (Table 4).

## Competing interests

The author declares that they have no competing interests.

## Supplementary Material

Additional file 1 **Figure S1: BensassonSI.pdf contains all the supplementary information for this study: Supplementary Text: Low error rate in Sanger Genome Resequencing Project and this study; Table S1: Primers used to amplify each centromere locus and for DNA sequencing; Table S2: Testing for evidence of recombination using LDhat and four-gamete analysis of centromeres in *S. cerevisiae*; Table S3: LDhat and four-gamete analysis of centromeres regions, but excluding centromeres themselves; Figure S1**. Centromeres and their immediate flanking DNA are too diverged to align *S. cerevisiae and S. paradoxus*; Figure S2. Centromeres and their immediate flanking DNA are highly diverged between *S. cerevisiae *and *S. mikatae*. Click here for file
