# Supplementary material for: Evidence for a high mutation rate at rapidly evolving yeast centromeres
Source: BMC Evol Biol. 2011 Jul 18;11:211. doi: 10.1186/1471-2148-11-211 (PMC3155921; doi:10.1186/1471-2148-11-211)
Supplement: Additional file 1 — Figure S1: BensassonSI.pdf contains all the supplementary information for this study: Supplementary Text: Low error rate in Sanger Genome Resequencing Project and this study; Table S1: Primers used to amplify each centromere locus and for DNA sequencing; Table S2: Testing for evidence of recombination using LDhat and four-gamete analysis of centromeres in S. cerevisiae; Table S3: LDhat and four-gamete analysis of centromeres regions, but excluding centromeres themselves; Figure S1. Centromeres and their immediate flanking DNA are too diverged to align S. cerevisiae and S. paradoxus; Figure S2. Centromeres and their immediate flanking DNA are highly diverged between S. cerevisiae and S. mikatae. [file 1471-2148-11-211-S1.PDF]

Supplementary Text

Low error rate in Sanger Genome Resequencing Project and this study

Light shotgun genome sequences for the 36 strains used here are available at one- to four-fold coverage as part of the Sanger Genome Resequencing Project (SGRP, [14]). The low coverage of these genome sequences prevented comprehensive sampling of centromeres from all SGRP strains and necessitated resequencing of all 16 centromeres from 36 strains of *S. cerevisiae*. However, the SGRP data provide a valuable resource for reciprocally estimating the sequencing error rate for the SGRP data and this study.

I compared 461,940 bp of unambiguous centromere sequence collected as part of this study to the corresponding 176,793 bp of SGRP sequence, where both datasets overlap and have quality scores of at least Q40. This comparison revealed a total of 10 discrepancies involving 3 strains and 3 centromere loci. PCR and re-sequencing revealed that the source of these errors is in the SGRP data. These results imply an error rate of approximately 1 in 18,000 bp for the SGRP data (~Q42) and < 1 in 180,000 bp for the centromere data collected here (>Q52). This error rate estimate extrapolates to an estimated < 2.61 sequencing errors in the total length of 461,940 bp *S. cerevisiae* centromere data in this study.

Estimated error rates for the SGRP data are consistent with the quality threshold of Q40 (< 1 in 10,000 bp) used, and the fact that these are low coverage shotgun data and so have quality values not very far above Q40 in many locations. In contrast, for centromere sequences collected here, targeted DNA sequencing of PCR products yielded data with at least an order of magnitude fewer errors for sites above the inclusion threshold of Q40.

Supp Table 1: Primers used to amplify each centromere locus and for DNA sequencing.

Locus	Primer Name	Primer Sequence	PCR and/or sequencing
cerC1	cerC1f	TTGTTTATTTCACTTTCGCGACT	pcr + seq
	cerC1r	AAAAAGAAGAAAAATAACTTTCTCATGTAA	pcr + seq
cerC2	cerC2f	TAAAGGGTTTGGAGCTGTTTTC	pcr + seq
	cerC2r	CGATGATTTTCAAGTAAAAGTGTTTG	pcr + seq
cerC3	cerC3f	CCAACAGATATAGGCTGTGTCTTAAA	pcr + seq
	cerC3r	ACAAGTCATGAATATGATAATGGTTACAC	pcr + seq
cerC4	cerC4f	ATATAGTGGTTGACATGCTGGCTA	seq
	cerC4f3	GGTGTGGAAGTCCTAATATCGG	pcr + seq
	cerC4r3	CCACCAATATCATTCTTTAGCAGTT	pcr
	cerC4r4	ATATATACTCCAGGTACAGTCCTCTAGGT	seq
cerC5	cerC5f	ATTTGAAACTTCACCAAAATCTTCTT	pcr + seq
	cerC5r	TTCTCATATATTGCACGTTCCG	pcr + seq
	cerC5f2	TTTCTGTCATTGAATGGCGA	seq
cerC6	cerC6f	CGAAGTTGTTAATGCTAAATACTCCA	pcr + seq
	cerC6r	TTTGTCTCTTAGAAGGTTTCTTTGGT	pcr + seq
cerC7	cerC7f2	GCTGCAGCATAATCTTTTCCTT	pcr + seq
	cerC7r2	ACGAATTTAGGTGCTACGCAA	pcr + seq
	cerC7f3	AAAGGTCCTGGAGAAAGCAA	seq
cerC8	cerC8f	CATATCATTTCATTTTGTTCTGGC	pcr + seq
	cerC8r	CCTTTAGAATTCTGGATTTGCG	pcr + seq
cerC9	cerC9f	ACCTAAAACAAGTAGGGTATTTGGAGT	pcr + seq
	cerC9r	CGTTAAAAGTTCTACCCAAGAAAAAG	pcr + seq
cerC10	cerC10f	AACGTTTGATATTCTACCCTGATGA	seq
	cerC10r	GGACTCAATGGTTCTTGCTCA	pcr + seq

	cerC10f3	TGTAGGTTCTTTGGCCATCC	seq
	cerC10f4	GCATTCGTACAGTTGTGTGCA	seq
	cerC10f5	GTGGTGTTATAAGAATGGTTGTTTTG	pcr
	cerC10r2	AAAAAMGCGATGAACAATAGC	seq
cerC11	cerC11f	GCTTGTAAATATATTGATCACGTGAAAT	pcr + seq
	cerC11r	GTTCTTGTATACGTGGAAGCTAGTCTATAA	pcr + seq
cerC12	cerC12f	AGGTACGATCATTTCCTTTTTAACC	pcr + seq
	cerC12r	TCGCAGTGTCAGTACCTCATAAGT	pcr + seq
cerC13	cerC13f2	GATGCCATCAATATTCGCCTA	pcr + seq
	cerC13r	ATTTACCCCTAGCATTAATACCCTTT	pcr + seq
cerC14	cerC14f	GAAGACGATGGTGAGGAGTAGATT	pcr + seq
	cerC14r	GGAGTTGGTAAAGAAAATCGAAAGTA	pcr + seq
cerC15	cerC15f	AAGGACGCACATATCTATAACAAGC	pcr + seq
	cerC15r	TTATCACCCTAGATAAAAGTACATCCG	pcr + seq
cerC16	cerC16f	AAGATGATTATAGGCGTATTATAGAGTCG	pcr + seq
	cerC16r	ATATATGTGCGCCCGGTAGAA	pcr + seq

Supp Table 2: Testing for evidence of recombination using LDhat and four-gamete analysis of centromeres in *S. cerevisiae*

Locus	θ_W	S_{LDhat}	Length	$4N_er$	R_{min}	P_{LPT}	P_{r2}	$P_{\|D'\|}$	S_{4G}	Muts.	P_h
CEN1	0.019	9	118	0.8	1	0.128	0.026	0.692	14	18	0.025
CEN2	0.017	9	131	2.7	3	0.42	0.397	0.384	21	25	0.206
CEN3	0.012	6	117	2.3	2	0.315	0.748	0.251	17	20	0.192
CEN4	0.015	7	117	0	3	0.813	0.933	0.919	14	18	0.033
CEN5	0.008	4	118	2.6	0	0.228	0.432	0.144	15	16	0.672
CEN6	0.019	9	118	0	0	0	1	1	14	15	0.622
CEN7	0.014	7	122	0	1	0.419	0.573	0.259	18	22	0.097
CEN8	0.021	10	118	0	0	0.262	0.598	0.539	20	20	1
CEN9	0.006	3	117	0	0	0	0.843	1	7	8	0.226
CEN10	0.006	3	123	0	0	0	0.662	0.671	12	14	0.172
CEN11	0.025	12	118	0	0	0.813	0.019	1	18	18	1
CEN12	0.006	3	120	0	0	0	0.651	1	15	17	0.316
CEN13	0.01	5	124	2.3	0	0.067	0.193	0.066	12	14	0.184
CEN14	0.018	9	119	0	0	0.021	0.025	0.064	15	16	0.642
CEN15	0.012	6	121	1.3	1	0.022	0.146	0.126	12	13	0.522
CEN16	0.008	5	143	4.0	1	0.055	0.079	0.638	10	11	0.409

The statistical significance of the evidence for recombination is shown by P_{LPT}, P_{r2} and $P_{|D'|}$, estimated by LDhat under a gene conversion model [21]. P_h is the probability that the estimated number of mutations at a locus (Muts.), are the result of homoplasy given the number of segregating sites in the four-gamete analysis (S_{4G}), and the length of DNA sequence. This probability is estimated from 1000 simulated replicates in R. Cells highlighted in yellow are significant at the 5% level, and this is consistent with number of recombination events estimated (R_{min} and $4N_er$). Cells highlighted in pink are show statistical

significant although this result is inconsistent with zero estimates of recombination events. Heterogeneity in the mutation rate within centromeres could result in this effect.

Supp Table 3: LDhat and four-gamete analysis of centromeres regions, but excluding centromeres themselves

Locus	θ_W	S_{LDhat}	Length	$4N_er$	R_{min}	P_{LPT}	P_{r2}	$P_{\|D'\|}$	S_{4G}	Muts.	P_h
CEN1	0.006	16	648	0	1	0.15	0.719	0.001	32	33	0.614
CEN2	0.009	31	827	0.6	3	0.014	0.686	0.142	64	73	0.003
CEN3	0.006	19	785	0.5	1	0.302	0.072	0.364	51	53	0.509
CEN4	0.005	15	743	0.5	1	0.151	0.005	0.733	34	35	0.572
CEN5	0.003	18	1397	0	0	0.451	0.651	0.481	48	48	1
CEN6	0.008	19	551	0.6	1	0	0.179	0.001	33	36	0.12
CEN7	0.006	10	403	0.8	1	0.342	0.566	0.803	24	25	0.555
CEN8	0.003	7	584	0.5	1	0.269	0.55	0.412	18	21	0.004
CEN9	0.002	4	535	5.2	1	0.818	0.111	0.955	19	20	0.312
CEN10	0.001	4	713	0.5	0	0.088	0.917	1	24	24	1
CEN11	0.009	27	700	0.6	2	0.004	0.095	0.004	46	51	0.023
CEN12	0.003	17	1255	0	0	0.684	0.065	0.782	52	52	1
CEN13	0.004	14	902	0.5	0	0.682	0.055	0.333	34	35	0.489
CEN14	0.001	8	1785	0.6	1	0.098	0.052	0.002	24	27	0.005
CEN15	0.004	9	597	0	0	0.865	0.141	0.07	30	30	1
CEN16	0.004	9	575	0.5	0	0.253	0.667	0.993	18	19	0.26

See Supp Table 2 legend for details.

Figure S1. Centromeres and their immediate flanking DNA are too diverged to align between *S. cerevisiae* and *S. paradoxus*. Centromeres for all 16 chromosomes of *S. cerevisiae* together with 2kb of flanking DNA from either side of the centromere, were compared to the corresponding regions in *S. paradoxus*. Exact centromere coordinates are shown in red. Dotplots were made using dottup (wordsize = 15), which is available as part of the EMBOSS package, and were then plotted in R. The reference genome sequences downloaded from SGRP were used for both *S. cerevisiae* and *S. paradoxus* (see Methods). The dotplot for CEN15 shows a repeat that includes the centromere in the reference sequence of *S. paradoxus* that is probably the result of missassembly of the reference strain sequence.

Figure S2. Centromeres and their immediate flanking DNA are highly diverged between *S. cerevisiae* and *S. mikatae*. The reference genome sequence for *S. mikatae* is published in Kellis et al (2003), [19]. Centromeres 3, 12 and 14 are omitted because in *S. mikatae* they occur in sequencing gaps.

Supp. Fig. 1

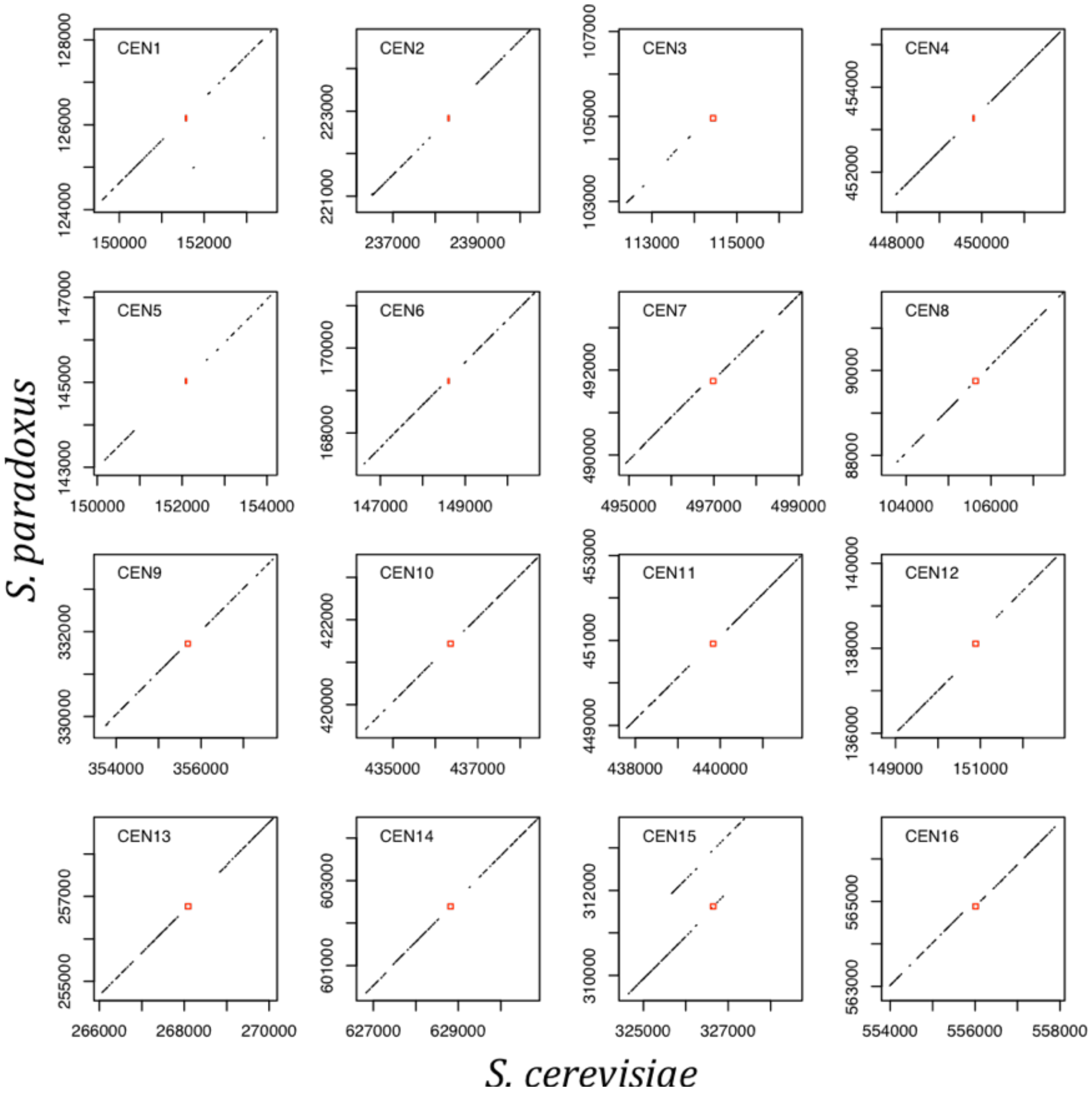
CEN1
CEN2
CEN3
CEN4
CEN5
CEN6
CEN7
CEN8
CEN9
CEN10
CEN11
CEN12
CEN13
CEN14
CEN15
CEN16
S. paradoxus
S. cerevisiae

Supp. Fig. 2

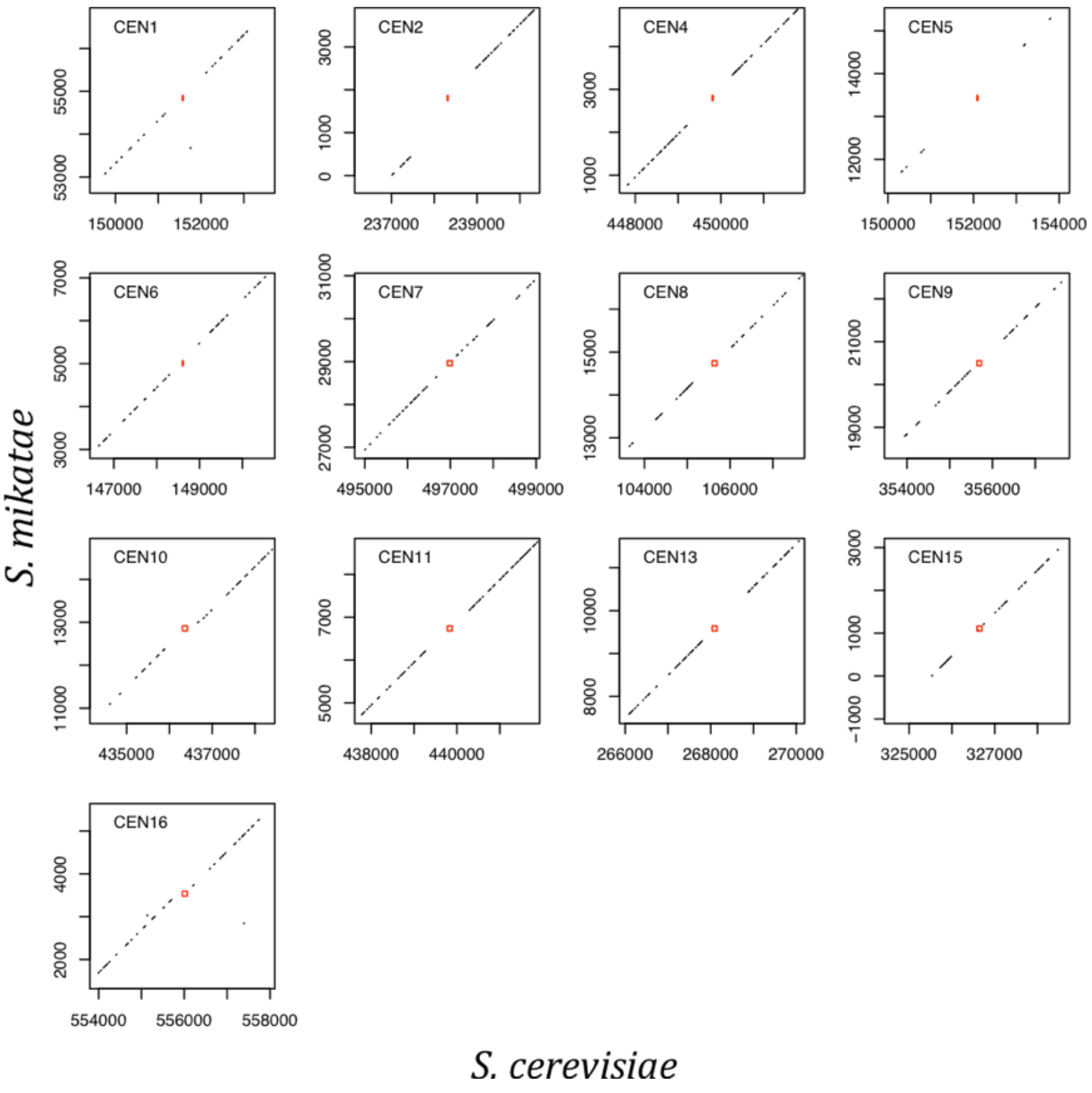
CEN1
CEN2
CEN4
CEN5
CEN6
CEN7
CEN8
CEN9
CEN10
CEN11
CEN13
CEN15
CEN16
S. mikatae
S. cerevisiae
